# Management of herpesvirus reactivations in patients with solid tumours and hematologic malignancies: update of the Guidelines of the Infectious Diseases Working Party (AGIHO) of the German Society for Hematology and Medical Oncology (DGHO) on *herpes simplex virus type 1*, *herpes simplex virus type 2*, and *varicella zoster virus*

**DOI:** 10.1007/s00277-021-04746-y

**Published:** 2022-01-07

**Authors:** Larissa Henze, Christoph Buhl, Michael Sandherr, Oliver A. Cornely, Werner J. Heinz, Yascha Khodamoradi, Til Ramon Kiderlen, Philipp Koehler, Alrun Seidler, Rosanne Sprute, Martin Schmidt-Hieber, Marie von Lilienfeld-Toal

**Affiliations:** 1 Department of Medicine, Clinic III - Hematology, Oncology, Palliative Medicine, Rostock University Medical Center, University of Rostock, Ernst-Heydemann-Str. 6, 18055 Rostock, Germany; 2 Department of Medicine, Clinic III – Oncology, Hematology, Immunoncology and Rheumatology/Clinical Immunology, University Hospital Bonn, Venusberg-Campus 1, 53127 Bonn, Germany; 3 Gemeinschaftspraxis für Hämatologie und Onkologie, 82362 Weilheim, Germany; 4 Faculty of Medicine and University Hospital Cologne, Department I of Internal Medicine, Excellence Center for Medical Mycology (ECMM), University of Cologne, Herderstraße 52, 50931 Cologne, Germany; 5 Faculty of Medicine and University Hospital Cologne, Cologne Excellence Cluster On Cellular Stress Responses in Aging-Associated Diseases (CECAD), University of Cologne, Herderstraße 52, 50931 Cologne, Germany; 6 German Centre for Infection Research (DZIF), Partner Site Bonn-Cologne, Herderstraße 52, 50931 Cologne, Germany; 7 Faculty of Medicine and University Hospital Cologne, Center for Integrated Oncology (CIO ABCD), University of Cologne, Herderstraße 52, 50931 Cologne, Germany; 8 Medical Clinic II, Caritas Hospital Bad Mergentheim, Uhlandstr, 7D-97980 Bad Mergentheim, Germany; 9 Department of Internal Medicine, Infectious Diseases, Goethe University Frankfurt, Theodor-Stern-Kai 7, 60590 Frankfurt Am Main, Germany; 10 Clinic for Hematology, Oncology, Palliative Medicine, Vivantes Klinikum Neukölln, Rudower Str. 48, 12359 Berlin, Germany; 11 Clinic for Hematology, Oncology and Tumor Immunology, Charité Universitätsmedizin Berlin, Campus Mitte Charitéplatz 1, 10117 Berlin, Germany; 12 Pharmaceutical Research Associates GmbH, Gottlieb-Daimler-Str. 10, 68165 Mannheim, Germany; 13 Privatpraxis München, 80992 München, Germany; 14 Department of Hematology and Oncology, Carl-Thiem-Klinikum Cottbus, Thiemstr. 111, 03048 Cottbus, Germany; 15 Department of Hematology and Medical Oncology, Clinic for Internal Medicine II, University Hospital Jena, Am Klinikum 1, 07747 Jena, Germany; 16 Leibniz Institute for Natural Product Research and Infection Biology, Hans Knöll Institute, Adolf-Reichwein-Str. 23, 07745 Jena, Germany

**Keywords:** Herpes stomatitis, Herpes zoster, Antiviral prophylaxis, Acyclovir, Solid tumours, Hematologic malignancies

## Abstract

Clinical reactivations of herpes simplex virus or varicella zoster virus occur frequently among patients with malignancies and manifest particularly as herpes simplex stomatitis in patients with acute leukaemia treated with intensive chemotherapy and as herpes zoster in patients with lymphoma or multiple myeloma. In recent years, knowledge on reactivation rates and clinical manifestations has increased for conventional chemotherapeutics as well as for many new antineoplastic agents. This guideline summarizes current evidence on herpesvirus reactivation in patients with solid tumours and hematological malignancies not undergoing allogeneic or autologous hematopoietic stem cell transplantation or other cellular therapy including diagnostic, prophylactic, and therapeutic aspects. Particularly, strategies of risk adapted pharmacological prophylaxis and vaccination are outlined for different patient groups. This guideline updates the guidelines of the Infectious Diseases Working Party (AGIHO) of the German Society for Hematology and Medical Oncology (DGHO) from 2015 “Antiviral prophylaxis in patients with solid tumours and haematological malignancies” focusing on herpes simplex virus and varicella zoster virus.

## Introduction

*Herpesviridae* persist — after primary infection usually in childhood or adolescence — lifelong in their hosts and can reactivate in situations of immune deficiency, like malignant diseases. Rates of reactivation depend on several factors such as underlying disease [1, 2], disease activity [3], antineoplastic therapy [1, 2], co-medication, comorbid conditions, and age [1]. Reactivation can lead to localized disease, as stomatitis and genital ulcers in case of *herpes simplex virus type 1* (HSV-1) and *type 2* (HSV-2) or herpes zoster in case of *varicella zoster virus* (VZV), but also to viral dissemination, cerebral or visceral disease contributing to significant morbidity and mortality [4, 5]. Different strategies to prevent symptomatic reactivation are possible: risk-adapted pharmacological prophylaxis, suppressive therapy (after severe complication or in case of multiple reactivations), and pre-emptive treatment (in asymptomatic patients after virus detection by screening methods) [6]. Additionally, to prevent herpes zoster vaccination has become available as general prophylaxis and is recommended for adults aged 50 years or older. The European Medicines Agency (EMA) has recently expanded the approval to adults ≥ 18 years who are at increased risk of herpes zoster.

Quantity of data as well as systematic reviews and present guidelines describe risks of herpesvirus reactivation and prophylactic interventions in patients undergoing allogeneic [6, 7] or autologous [8] hematopoietic stem cell transplantation (HSCT). However, systematic analyses and trials of herpesvirus reactivation in patients with solid tumours or hematologic malignancies who are not candidates for HSCT are limited [4] but increasing, acknowledging the variable risks in the era of new therapeutics. This guideline summarizes current evidence on herpesvirus reactivation in patients with solid tumours and hematologic malignancies not undergoing allogeneic or autologous HSCT or cellular therapy (CAR T cell therapy) and constitutes an update of the guideline of the Infectious Diseases Working Party (AGIHO) of the German Society for Hematology and Medical Oncology (DGHO) of 2015 “Antiviral prophylaxis in patients with solid tumours and hematological malignancies” [9] focusing on HSV-1, HSV-2, and VZV.

## Methods

In 2015, recommendations for antiviral prophylaxis in patients with solid tumours and hematologic malignancies were published by the AGIHO [9]. Meanwhile, many new antineoplastic drugs have been approved. Drug combinations are used frequently for the treatment of patients with solid tumours and hematological malignancies. Moreover, vaccination has become available as preventive strategy for VZV reactivation. Therefore, an update of the recommendations was deemed necessary. This article represents the update regarding HSV-1, HSV-2, and VZV; recommendations regarding *Epstein-Barr virus* (EBV), *cytomegalovirus* (CMV), and *human herpesvirus type 6* (HHV-6) will be updated in a separate article.

An expert panel of twelve oncologists, infectious disease specialists, and microbiologists — all members of the AGIHO — conducted independent literature search of the PubMed and Medline databases, using the following search terms: herpes simplex cancer prophylaxis, herpes simplex cancer therapy, varicella zoster cancer prophylaxis, and varicella zoster cancer therapy, restricted to adult patients. The literature search extended initially from January 2013 to August 2020, and was updated on 30 April 2021. Articles in English and German were included. References and other literature published before 2013 were included in the assessment process if relevant. Abstracts presented at the annual meetings of the DGHO, the European Hematology Association (EHA), the European Society of Medical Oncology (ESMO), the American Society of Hematology (ASH), and the American Society of Oncology (ASCO) from 2013 to 2020 were included if relevant. The expert panel weighed the search results in a stepwise consensus process consisting of personal meetings, video conferences, and e-mail discussions. Strength of recommendation (SoR) and quality of evidence (QoE) were graded according to the criteria applied by the European Society for Clinical Microbiology and Infectious Diseases (ESCMID) [10] (Table 1). A formal consensus meeting took place at the general assembly of the AGIHO, as video conference on 6 October 2020, to which all members of the AGIHO were invited. Recommendations were finally approved by discussion and online voting among the participating experts. This guideline provides an evaluation of present evidence (which is sparse in some fields) and the experts’ consensus interpretation. The recommendations intend to assist physicians in decisions on individual patients [8].

**Table 1:** Strength of recommendation (SoR) and quality of evidence (QoE) as proposed by the European Society of Clinical Microbiology and Infectious Diseases [10]

Category	Definition
Strength of recommendation (SoR)	
A	Strongly supports a recommendation for use
B	Moderately supports a recommendation for use
C	Marginally supports a recommendation for use
D	Supports a recommendation against use
Quality of evidence (QoE)—level	
I	Evidence from at least one properly designed randomized, controlled trial
II	Evidence from at least one well-designed clinical trial, without randomization; from cohort- or case-controlled analytic studies (preferably from > 1 center); from multiple time series; or from dramatic results of uncontrolled experiments
III	Evidence from opinions of respected authorities, based on clinical experience, descriptive case studies, or reports of expert committees
Quality of evidence (QoE) – index, for level II	
r	Meta-analysis or systematic review of randomized controlled trials
t	Transferred evidence, that is, results from different patient cohorts, or similar immune-status situation
h	Comparator group is a historical control
u	Uncontrolled trial
a	Published abstract (presented at an international symposium or meeting)

## Manifestations of HSV-1, HSV-2, and VZV

After primary infection, *herpesviridae* establish latency in sensory neural ganglia [11] (Table 2). Reactivation can occur by several triggers in the healthy population, but particularly in situations with reduced cell-mediated immunity [12]. Risk of symptomatic reactivation increases with intensity and duration of functional T cell suppression [4, 11].

**Table 2:** Neurotrophic latency and forms of reactivation of herpes simplex virus type 1 (HSV-1), herpes simplex virus type 2 (HSV-2), and varicella zoster virus (VZV)

	HSV-1	HSV-2	VZV
Neurotrophic latency	Ganglion trigeminale, ganglion sacrale	Ganglion sacrale, Ganglion trigeminale	Cranial nerve ganglia, dorsal root ganglia
Reactivation	Asymptomatic viral shedding Herpes labialis Stomatitis^a)^ Herpes genitalis Oesophagitis ^a)^ Hepatitis ^a)^ Colitis ^a)^ Pneumonitis ^a)^ Encephalitis Keratitis	Asymptomatic viral shedding Herpes genitalis Hepatitis ^a)^ Meningitis Encephalitis	Herpes zoster ^b)^ Disseminated herpes zoster ^a)^ Hepatitis ^a)^ Pancreatitis ^a)^ Pneumonitis ^a)^ Meningoencephalitis Cerebral vasculopathy Keratitis, uveitis, retinitis

^a^In immunocompromised patients.

^b^Including atypical herpes zoster and zoster sine herpete (often presenting as visceral zoster).

Primary infection with HSV-1 occurs mainly in the oropharyngeal mucosa, but is unrecognized or asymptomatic in more than 80% of individuals [13, 14]. On the contrary, primary infection can be severe, manifesting as encephalitis, particularly in the immunocompromised individual. Although seroprevalence of HSV-1 reaches 90% in the general population by the age of 50 years [14, 15], only about one-third will suffer from symptomatic reactivation during lifetime [14]. Symptomatic reactivation typically occurs as herpes labialis (“coldsores”), but in the immunocompromised person, herpes stomatitis is often seen [12] (Table 2). Life-threatening reactivations are HSV encephalitis, HSV pneumonitis, and rarely other visceral manifestations [5, 16–18] (Table 2). Besides, HSV-1 is increasingly found in genital herpes [19] (Table 2). HSV-1 can also be detected in oral swabs in asymptomatic or oligosymptomatic individuals [14, 20].

Acquisition of HSV-2 results in infection at genital, perigenital, or anal skin sites, with seeding to sacral ganglia [21]. Occurrence in the oropharyngeal mucosa is less frequent in HSV-2. Severe primary manifestations are meningitis or other organ disease, mainly at very young age or in the immunocompromised. Seroprevalence shows considerable variation in different populations and ranges from 15 to 25% in industrialized countries [22]. Symptomatic reactivations mostly lead to genital ulcerous lesions and occur frequently [19]. Asymptomatic viral shedding is also common [23]. Severe manifestations are similar to HSV-1 (Table 2).

Primary infection with VZV in the naïve population results in varicella disease (“chickenpox”). Besides the characteristic skin lesions of macules, which progress to papules and then vesicles, systemic symptoms, like fever, may be present and are more pronounced in adolescents or adults compared to children [11]. Primary infection with VZV can affect organs and be life-threatening if it occurs in the immunocompromised individual. VZV establishes latency in sensory neurons and commonly reactivates as herpes zoster (“shingles”) in the elderly (≥ 50 years old), due to waning T cell immunity [11, 24, 25] (Table 2). While the incidence rate of herpes zoster in the population is 3.2 cases per 1000 person-years [26], it increases with age to 9.1 per 1000 person-years among persons 50 years of age or older [27]. Likewise, other conditions of T cell suppression may lead to herpes zoster in the younger population [24]. The incidence rate of herpes zoster is reported to be between 12 per 1000 person-years in individuals with solid tumours receiving immunosuppressive chemotherapy and 31 per 1000 person-years in patients with hematological malignancies receiving treatment [28, 29]. Typical herpes zoster appears as vesicular lesions in the distribution of dermatomes, accompanied by neuropathic pain [11], which can become chronic, leading to post-herpetic neuralgia in 15% [24]. Herpes zoster affecting the cranial nerves V (zoster ophthalmicus) or VII/VIII (zoster oticus or Ramsay Hunt syndrome) can be followed by longterm visual or hearing dysfunction. Further manifestations of reactivation in immunocompromised people are disseminated herpes zoster, meningoencephalitis, cerebral vasculopathy, pneumonitis, hepatitis, pancreatitis, or visceral zoster, that might be misdiagnosed as acute abdomen [11, 24, 30–32] (Table 2). Because VZV is highly contagious, > 90% of people have become infected before adolescence prior to widespread implementation of vaccination [24]. However, since vaccination against varicella during early childhood with a live attenuated varicella virus (vOka) has become universal practice in Germany in 2004 like in several countries [24, 25], the younger generation will not have experienced varicella disease, but the majority will have been vaccinated. Reactivation of the vaccine-type VZV (vOka) has been seen sporadically [24].

Studies on prophylactic strategies used different definitions of viral manifestation [33, 34] and focused on different endpoints: clinical manifestations (without virological confirmation) or clinical manifestations with virological findings or virus detection. Mortality is only reported in few studies. Whereas the terms *disease*, *infection*, and *reactivation* are not used consistently throughout literature, we will nominate primary manifestations as either *infection (asymptomatic)* or *disease (symptomatic)*. Primary manifestations rarely occur during cancer treatment. By contrast, *reactivations* are frequent in cancer patients, particularly while on tumour treatment [35] and can be asymptomatic (*viral shedding*) or symptomatic, thus to be named here as *disease by reactivation (clinical reactivation)* (Table 2). In the immunocompromised patient, the clinical picture may be severe [5, 11, 21, 36] contributing to morbidity and mortality (Table 2). Risk-dependent prophylactic strategies are warranted to reduce the rate of clinical reactivation (e.g. stomatitis, herpes zoster, fever), complications (e.g. dissemination, post-herpetic neuralgia) and mortality. These are summarized in this guideline.

## Diagnostics

### Serology

Serology testing for HSV-1, HSV-2, and VZV is useful to ascertain prior infection in patients with acute leukaemia undergoing intensive chemotherapy and therefore identify patients at risk of reactivation (BIII) [11, 37] (Tables 3 and 4). However, seroprevalence of HSV-1 and VZV is about 90% for adults [14, 24], and thus, the majority of patients will have to be considered at risk of reactivation. Therefore, a universal prophylactic strategy for all patients with indication for prophylaxis of HSV-1 or VZV can be an alternative approach.

**Table 3:** Recommendations for diagnostics of herpes simplex virus (HSV — if not otherwise specified referring to HSV-1 and HSV-2). Yield of all tests for virus detection might be influenced by whether the patient is receiving antiviral prophylaxis or not

Clinical situation	Intention	Diagnostic strategy	SoR	QoE	Comments	Reference
Patients at risk of HSV reactivation (patients with acute leukaemia planned for intensive therapy or other specified patient group)	Diagnosis of prior exposure, to decide about prophylaxis ^a)^	HSV serology (IgG)	B	III	(see text)	[11, 37]
Patients with suspicion of HSV disease	To diagnose HSV disease	HSV serology (IgM, serial IgG)	D	III	Low sensitivity, time delay	[11]
	To diagnose HSV disease	qPCR for HSV vs. viral culture (mucosal swab, BW, BAL)	A	IItu	qPCR with higher sensitivity, reliability, speed	[39, 47, 48]
Patients with stomatitis after (radio-) chemotherapy	To diagnose HSV stomatitis	qPCR for HSV-1 (oral swab)	C	IIu		[14, 20, 43]
Patients with clinical diagnosis of herpes genitalis	To diagnose HSV	qPCR for HSV (genital or perianal swab, preferably vesicle content)	A	III	For differential diagnosis	[19, 44]
Patients suspected for herpes encephalitis	To diagnose HSV encephalitis	qPCR for HSV (CSF)	A	IItu	No exclusion by negative result, particularly if therapy has already started	[42, 49]
	To diagnose HSV encephalitis	HSV IgG (CSF/serum)	C	III	Additionally	[11, 49]
Patients suspected for herpes pneumonitis	To diagnose HSV pneumonitis	qPCR (BW, BAL)	A	IIu	HSV DNA may also stem from oropharyngeal sites (see text)	[48, 50]
Patients suspected for other organ HSV disease	To diagnose HSV visceral disease	qPCR for HSV (organ biopsy)	A	IItu	No exclusion by negative result, particularly if therapy has already started	[48]
Asymptomatic patients at risk for HSV reactivation	To screen for viral replication	qPCR for HSV-1 (mucosal swab)	D	I	Asymptomatic viral shedding; pre-emptive treatment not recommended	[11, 51]

*BAL* bronchoalveolar lavage, *BW* bronchial wash, *CSF* cerebrospinal fluid, *QoE* quality of evidence, *qPCR* quantitative polymerase chain reaction, *SoR* strength of recommendation.

^a^We consider a universal prophylactic strategy for all patients with indication for prophylaxis of HSV as equally appropriate, because seroprevalence of HSV-1 is about 90% for adults.

**Table 4:** Recommendations for diagnostics of varicella zoster virus (VZV). Yield of all tests for virus detection might be influenced by whether the patient is receiving antiviral prophylaxis or not

Clinical situation	Intention	Diagnostic strategy	SoR	QoE	Comments	Reference
Patients at risk of VZV reactivation (patients with lymphoma or multiple myeloma or other specified patient group)	Diagnosis of prior exposure, to decide about prophylaxis^a^	VZV serology (IgG)	B	III		
Patients with suspicion of VZV disease	To diagnose VZV disease	VZV serology (IgM, serial IgG)	D	III	Low sensitivity, time delay	[40, 52]
	To diagnose VZV disease	qPCR for VZV versus DFA or viral culture (skin swab, vesicle content)	A	IItu	qPCR with higher sensitivity, reliability; qPCR applicable on varying specimen	[41, 52]
Patients with typical segmental zoster lesion	To diagnose VZV	qPCR for VZV (skin swab)	C	III	Usually diagnosis on clinical grounds; for differential-diagnosis to HSV	[52]
Patients with atypical zoster lesion	To diagnose herpes zoster	qPCR for VZV (skin swab)	A	III		[52]
	To diagnose herpes zoster	qPCR for VZV (saliva)	B	II	Saliva more sensitive than blood	[40, 45]
	To diagnose herpes zoster	qPCR for VZV (blood)	C	II		[40, 45]
Patients with suspected zoster sine herpete	To diagnose VZV disease	qPCR for VZV (blood)	A	II	For rapid diagnosis	[40, 45, 52]
	To diagnose VZV disease	qPCR for VZV (saliva)	B	II		[40, 45]
Patients with suspected disseminated zoster	To diagnose VZV disease	qPCR for VZV (blood)	A	III	Not necessary if clinical diagnosis is obvious	[52]
Patients with zoster ophthalmicus	To diagnose ocular involvement	qPCR for VZV (affected superficial structure of the eye)	A	III	Ophthalmological examination recommended and often sufficient for diagnosis	[52]
Patients suspected for VZV encephalitis	To diagnose VZV encephalitis	qPCR for VZV (CSF)	A	II	No exclusion by negative result, particularly if therapy has already started	[49, 52, 53]
	To diagnose VZV	VZV IgG (CSF/serum)	C	III	Alternative in cerebral vasculopathy	[40, 49, 52]
	To diagnose VZV	qPCR for VZV (blood)	C	II		[53]
Patients suspected for VZV pneumonitis	To diagnose VZV pneumonitis	qPCR for VZV (BAL)	A	IIu	No exclusion by negative result, particularly if therapy has already started	[54]
	To diagnose VZV	qPCR for VZV (blood)	B	IIu		[54]
Patients suspected for other organ VZV disease	To diagnose VZV visceral disease	qPCR for VZV (organ biopsy)	A	III	No exclusion by negative result, particularly if therapy has already started	[11]
	To diagnose VZV	qPCR for VZV(blood)	A	III	No exclusion by negative result, particularly if therapy has already started	[52, 55]
Asymptomatic patients at risk for VZV reactivation	To screen for viral replication	qPCR for VZV (blood)	D	III	Pre-emptive treatment not recommended	[11]

*BAL* bronchoalveolar lavage, *CSF* cerebrospinal fluid, *DFA* direct fluorescence antibody, *HSV* herpes simplex virus, *IgG* immunoglobulin G, *QoE* quality of evidence, *qPCR* quantitative polymerase chain reaction, *SoR* strength of recommendation, *VZV* varicella zoster virus.

^a^We consider a universal prophylactic strategy for all patients with indication for prophylaxis of VZV as equally appropriate, because seroprevalence of VZV is about 90% for adults.

Serology testing (IgM or serial IgG) is not recommended to diagnose primary infection or reactivation of HSV or VZV due to low sensitivity [11], possible cross-reaction [38], and time delay (DIII) (Tables 3 and 4).

### Virus detection

Quantitative polymerase chain reaction (qPCR) is a specific and sensitive technique for the detection of HSV-1, HSV-2 and VZV DNA. It is more reliable than direct virus antigen detection and viral culture, and also faster [11, 19, 39–41]. Moreover, the technique can be applied on different material, such as swabs (e.g. oral, genital, perianal, affected skin), vesicle content, blood, saliva, intraocular fluid, bronchial wash (BW), bronchoalveolar lavage (BAL), cerebrospinal fluid (CSF), and biopsies [38]. Thus, HSV/VZV qPCR is the method of choice to diagnose infection or reactivation (AII) (Tables 3 and 4).

Because HSV qPCR is as sensitive as detecting copy numbers of viral DNA at 10/sample [11] or 10/mL [42], the question arises whether a positive qPCR result of HSV necessarily means viral reactivation, or whether it represents an accidental finding of a small quantity of virus genome that probably always exists in latency [14]. Considering that HSV has a lytic effect on epithelial cells, the subclinical excretion of the virus is connected with microscopically visible or invisible ulcerations that remain unnoticed by the patient and the physician, a situation termed *asymptomatic viral shedding*, but still virus replication thus reactivation [14]. Therefore, it is not possible to define a threshold for diagnosis of *HSV disease by reactivation* at mucous or cutaneous sites [38, 43]. Disease by reactivation might be more likely with higher copy numbers of HSV DNA [20, 39]. By contrast, detection of HSV in CSF or biopsies is clinically significant [11]. Of note, negative PCR results cannot be interpreted as exclusion of diagnosis of HSV disease [44]. SoR and QoE for performing viral diagnostic for HSV-1 and HSV-2 in different clinical situations are listed in Table 3.

Diagnosis of primary VZV disease or VZV disease by reactivation can usually be made on clinical appearance as varicella and herpes zoster. Still, vesicle formation is a hallmark of VZV and HSV [45], and analysing VZV qPCR (swabs from affected skin is sufficient) and HSV qPCR (vesicle content is optimal) may be indicated to evaluate the causative agent, e.g. for assessment of transmission risk. VZV DNA in blood can be detected in varicella preceding the occurrence of rash by about ten days and persisting for two to three weeks. VZV DNA in blood is usually found at the onset of herpes zoster and for many weeks thereafter [40]. VZV DNA is also found in oropharynx, making saliva a suitable material for qPCR analysis of VZV [40, 46]. Therefore, VZV qPCR of blood or saliva can be helpful, particularly for atypical manifestations [24, 40] or when visceral disease is suspected in the absence of pathognomonic skin lesions [11]. Reported sensitivity of VZV DNA is 20–400 copies/mL depending on material [40]. SoR and QoE for performing viral diagnostics in different clinical situations with suspicion of VZV disease are listed in Table 4.

In asymptomatic patients at risk, there is no indication for screening and pre-emptive therapy of HSV-1, HSV-2, or VZV (DI; DIII) (Tables 3 and 4) [40]. Clinical suspicion should prompt immediate treatment as well as further diagnostics, including tissue biopsies, if appropriate [11] (Tables 3 and 4). Emphasis must be placed on clinical awareness of HSV or VZV reactivations in immunocompromised patients [13]. The absence of mucous or cutaneous lesions does not rule out the possibility of HSV and VZV disease since manifestations can be atypical.

### Testing of resistance

Clinical reactivation of HSV or VZV in patients on antiviral prophylaxis does not imply routine resistance testing. Incompliance [56], omitted medication for other reasons [57] and reduced oral bioavailability [58] have to be considered. In contrast, testing for resistance has to be considered in patients on treatment with acyclovir, if no clinical improvement is seen after 5 days [38]. Whereas acyclovir resistance in VZV is a rarity, resistance in HSV is occasionally observed, necessitating a switch in antiviral treatment. Genotypic resistance testing is recommended and established in specialized laboratories.

### Contribution of imaging techniques to diagnosis

Besides high awareness for clinical signs in populations at risk for HSV or VZV reactivation, imaging can be indicative in some manifestations: Cranial magnetic resonance imaging (MRI) is recommended for diagnosing encephalitis by HSV/VZV or cerebral vasculopathy in VZV and may be helpful in conjunction with other methods such as CSF PCR [49]. Pulmonary infiltration detected by chest computed tomography (CT) can indicate HSV or VZV pneumonitis, even though the appearance is not specific. On bronchoscopy, rendering a confident diagnosis of HSV or VZV pneumonitis is challenging, as many patients lack easily detectable herpetic lesions and findings might be nonspecific by superimposed coinfections [48].

## Pharmacological prophylaxis

Acyclovir has for long been the mainstay for prophylaxis and treatment of HSV and VZV in immunocompromised patients [21].

A systematic review of oral herpetic viral disease of the Multinational Association of Supportive Care in Cancer (MASCC)/International Society of Oral Oncology (ISOO) showed that acyclovir orally is effective in preventing oral herpetic viral disease in patients with solid tumours or hematologic malignancies [34]. A most recent network meta-analysis compared different acyclovir regimens, evaluated in thirteen randomized controlled trials (RCT) on prevention of oral HSV disease in patients undergoing cancer treatment, including allogeneic or autologous HSCT: oral acyclovir 400 mg four times daily (QID) or oral acyclovir 400 mg twice daily (BID) or intravenous acyclovir 250 mg/m^2^ TID were identified as most effective regimens to prevent oral HSV disease [36]. Since oral acyclovir 400 mg BID had an almost similar activity to oral acyclovir 400 mg QID, clinicians might prefer prescribing acyclovir 400 mg BID in most patients, particularly if renal function is a concern [36]. Valacyclovir, the L-valyl ester of acyclovir, has better oral bioavailability [36, 59]. Valacyclovir has been shown to be non-inferior to acyclovir in preventing HSV stomatitis in patients with acute leukaemia and intensive chemotherapy [60]. However, due to the limited number of reported trials, evidence is low and a network meta-analysis was not possible [36].

We recommend oral acyclovir 400 mg BID or 400 mg QID as prophylaxis of *oral HSV disease* (AIIr). Oral valacyclovir can be used alternatively (BI), but the best regimen (250 mg BID or 500 mg BID) has not yet been defined. For patients not tolerating oral medication, intravenous acyclovir 250 mg/m^2^ TID is suitable (AIIr).

Long-term (6 to 12 months) oral administration of acyclovir, valacyclovir, or famciclovir, a well-absorbed prodrug of penciclovir, suppresses genital herpes in patients who have frequent recurrences (suppressive therapy). In a Cochrane meta-analysis of 26 trials in patients with at least four recurrences of genital herpes per year, 6950 participants were randomly assigned to either of the antiviral drugs or placebo [22]. Patients with immunosuppression were excluded in most of the trials [22]. Clinical recurrence of genital herpes was reduced with acyclovir (nine trials, pooled RR 0.48), valacyclovir (four trials, pooled RR 0.41), or famciclovir (two trials, pooled RR 0.57) [22]. The network meta-analysis was unable to determine which of the drugs was most effective [22, 23].

Thus, acyclovir, valacyclovir, and famciclovir have all shown to be effective in reducing the risk of *recurrent genital herpes* and of asymptomatic viral shedding. Acyclovir 400 mg BID has been tested and used widely (AI); valacyclovir 500 mg BID and famciclovir 500 mg BID are approved for this indication in immunocompromised patients (BIIt).

Acyclovir has been shown to reduce clinical VZV reactivation and to increase survival in patients undergoing HSCT, for whom the reactivation risk of VZV is as high as 50% [35]. Thus, antiviral prophylaxis with acyclovir has become standard of care in allogeneic and autologous HSCT recipients [4, 6, 8, 38, 61]. VZV reactivation risk in non-HSCT patients undergoing tumour treatment is also elevated, particularly in patients with lymphoma or multiple myeloma and best described for patients with multiple myeloma treated with proteasome-inhibitors, being up to 15% without antiviral prophylaxis [62]. VZV reactivation has remarkable influence on morbidity and quality of life. Therefore, antiviral prophylaxis is standard of care in these patients (for recommendations see below). However, no randomized controlled trials have been performed to find the most effective regimen (acyclovir has been used as 400 mg orally once daily, BID or TID most widely) or to compare acyclovir to valacyclovir or famciclovir.

We recommend oral acyclovir to reduce clinical *VZV reactivation*; dosages from 400 mg once daily to 400 mg TID have been shown to be effective (AII). Valacyclovir may also be effective by mechanism of action and due to transferred evidence, but has rarely been systematically tested [63, 64] (CIIu). For patients not tolerating oral medication, intravenous acyclovir is suitable, but evidence on the most appropriate dosage is lacking (BIIt).

Of note, all three nucleoside analogues (acyclovir, valacyclovir, famciclovir) require dose adjustment in patients with renal impairment.

## Vaccination

VZV is the only human herpesvirus for which highly effective vaccines are available [24]. Since the introduction of a live attenuated varicella virus (vOKA) during childhood as primary prophylaxis against VZV, varicella disease and severe primary manifestations have decreased [25]. Still, the adult population nowadays almost universally experienced varicella disease and is therefore at risk of VZV reactivations, and incidences of VZV reactivation are rising. Recently, two different types of herpes zoster vaccines have been studied and approved [26]: The live-attenuated zoster vaccine Zostavax® is contraindicated in the immunocompromised [26]. The adjuvanted recombinant (non-live) subunit zoster vaccine Shingrix® has recently been approved for the prevention of herpes zoster and post-herpetic neuralgia for adults 50 years of age or older and for adults of any age at increased risk of herpes zoster. It has been studied in specific groups of patients with malignancies and shown to be safe [26, 65–68]. Nevertheless, immunogenicity [26, 66–68] and efficacy rates [26] seemed lower in patients while on tumour treatment than in the general elderly population: For instance, the incidence rate of herpes zoster was reduced from 66.2 per 1000 patient-years (placebo group) to 8.5 per 1000 patient-years (vaccine group) in 562 adults (≥ 18 years old) with hematologic malignancies in the zoster-39 trial [26], whereas in the ZOE-50 trial, herpes zoster incidence per 1000 patient-years was reduced from 9.1 (placebo group) to 0.3 (vaccine group) in more than 13.000 persons of at least 50 years of age [27]. Considering herpes zoster, vaccination with the recombinant zoster vaccine Shingrix® is recommended due to safety and immunogenicity, although data on clinical efficacy in certain malignancies are preliminary [66, 67, 69] and long-term protection rates are sparse. Until more data are available, particularly of comparative trials for vaccination versus pharmacological prophylaxis, it is suggested to apply acyclovir additionally in high risk patient groups (for recommendations see below). Around 30% of participants of the zoster-39 trial (i.e. patients with hematologic malignancies) received antiviral prophylaxis [26]. Nevertheless, it is anticipated that an increased use of the recombinant zoster vaccine Shingrix® in immunosuppressed patients would — if clinically shown effective — lead to a decreased use of pharmacological prophylaxis [70].

## Patient groups

### Patients with solid tumours

Compared to patients with hematologic malignancies with cumulative incidences of herpesvirus reactivations of up to 20% during the treatment phase and 5-year follow up, frequencies of herpesvirus reactivations are low (< 10%) in patients with solid tumours [4]. However, case reports and case series about severe complications by HSV or VZV have been described [5, 16–18]. This holds also true for patients treated with immune checkpoint inhibitors with or without chemotherapy [71, 72]. An intrinsically increased risk of clinical reactivation of HSV and VZV for immune checkpoint inhibitor therapy has not been described [73, 74]. But an increased risk has been seen with immunosuppressive treatments (corticosteroids and others), as used frequently in case of immune related adverse events [74]. Depending on dose and duration of immunosuppressive treatments antiviral prophylaxis may be warranted (Table 5).

**Table 5:** Recommendations for pharmacological prophylaxis in patients with solid tumours

Clinical situation	Intention	Intervention	SoR	QoE	Comments	Reference
Patients with solid tumours and systemic therapy (in general; for specific risks see below)	To prevent HSV/VZV reactivation	Acyclovir	D	III	Low risk of reactivation	
Patients with HNSCC, treated with radiochemotherapy	To prevent HSV stomatitis	Acyclovir	C	IIr		[34]
Patients with malignancies, taking corticosteroids in high doses long term (> 10 mg PEQ per day for 14 days or longer)	To prevent herpes zoster	Acyclovir	C	IIu	Persisting risk for several months after corticosteroid has been stopped (see text)	[77]

In patients with normal renal function, acyclovir is recommended with 400 mg orally BID (for more details refer to section “Pharmacological Prophylaxis).

*HNSCC* head and neck squamous cell carcinoma, *HSV* herpes simplex virus, *PEQ* prednisolone equivalent, *QoE* quality of evidence, *SoR* strength of recommendation, *VZV* varicella zoster virus.

The following patient groups need particular attention for HSV disease by reactivation (Table 5):

HSV stomatitis can occur and be severe in patients with head and neck cancer treated with radiochemotherapy [14]. Irradiation contributes to regional eradication of the cellular immune component that is responsible for controlling herpesvirus latency [75]. While no general recommendation on pharmacological prophylaxis can be given due to lack of evidence, acyclovir and valacyclovir have been shown to be preventive in an updated meta-analysis of 41 trials in different patient populations including patients with head and neck cancer treated with radiochemotherapy [34] (CIIr). Likewise encephalitis by HSV-1 has been described in patients with cranial irradiation of metastases [5, 17] and for malignant glioma [16]. In malignant glioma, concomitant treatment with dexamethasone and temozolomide increases the risk for HSV encephalitis [16]. To our knowledge, no trials have analysed the efficacy of pharmacological prophylaxis for this situation. Clinical awareness is important.

For disease by reactivation of VZV, specifically herpes zoster, the age and gender standardized incidence in relation to the general population is 4.8 for hematologic malignancies and 1.9 for solid tumours [28]. At 5 years, the cumulative incidence of herpes zoster is low at 5% in patients with solid tumours without antiviral prophylaxis [28]. Pharmacological prophylaxis to prevent disease by reactivation of VZV in patients with solid tumours is not generally indicated (DIII) (Table 5). Corticosteroids are used as supportive care in patients with solid tumours and hematologic malignancies. Dosages of 10 mg prednisolone equivalent (PEQ) or more per day for at least 14 days have been described to increase the risk of VZV reactivation [76]. In a large population-based cohort study of patients with different diseases (cancer, asthma, autoimmune diseases), the HR for herpes zoster was 2.37 compared to a matched population not taking corticosteroids [77]. The risk increased with concomitant immunosuppressive medication [77]. Therefore, even though no evidence from randomized trials exists, it might be reasonable to consider patients on corticosteroids, particularly with high doses long term (and/or if concomitantly treated or heavily pre-treated with immunosuppressive agents) for antiviral pharmacological prophylaxis (CIIu) (Table 5). Antiviral prophylaxis might be continued for up to 6 months after corticosteroids have been terminated [76].

### Patients with acute leukaemia

The incidence of clinical reactivation of HSV in patients with acute leukaemia treated with intensive chemotherapy is high, affecting 37–68% of patients [14, 33]. In the majority, ulcerative stomatitis is seen, less commonly oesophagitis, pneumonitis, or genital lesions [60]. A systematic review and meta-analysis of antiviral prophylaxis in patients with hematologic malignancies performed by Yahav et al. [33] identified 22 trials, most of them referring to patients with allogeneic or autologous HSCT. Five trials included patients with intensive chemotherapy [33, 78–82]. Rates of symptomatic HSV reactivation were significantly lower with acyclovir compared to placebo (RR 0.10) [33]. There was no significant difference in overall mortality (RR 1.27) [33]. Even though the respective trials have all been performed more than twenty years ago (and particularly diagnostic methods as well as supportive care have evolved since then), our literature search could not reveal new trials to be considered, in line with the results of a very recent network meta-analysis by Aribi Al-Zoobaee et al. [36]. Considering the high risk of reactivation prophylactic acyclovir or valacyclovir should be applied in patients with acute leukaemia receiving intensive chemotherapy to reduce HSV stomatitis, and to reduce other HSV reactivations (BIIr) (Table 6). In patients with acute myeloid leukaemia antiviral prophylaxis should start with the initiation of intensive remission induction chemotherapy and be continued during the neutropenic phase. However, the duration varied from 28 to 100 days in clinical studies. No data specifically on consolidation therapy are available, but the incidence of oral HSV disease was much lower [20].

**Table 6:** Patients with acute leukaemia and myeloproliferative neoplasms

Clinical situation	Intention	Intervention	SoR	QoE	Comments	Reference
Patients with AML/high-risk MDS, planned for intensive therapy	To prevent HSV stomatitis and other clinical manifestations of HSV	Acyclovir, valacyclovir^a^	B	IIr	For remission induction chemotherapy (see text)	[33, 36, 60, 79, 129]
	To prevent herpes zoster (and other clinical reactivation of VZV)	Acyclovir, valacyclovir^a^	B	IIr	Particularly in patients with APL treated with arsenic trioxide (see text)	[33, 83]
Patients with ALL	To prevent HSV stomatitis and other clinical manifestations of HSV	Acyclovir	B	I	While on treatment	[78, 81]
	To prevent herpes zoster^b^	Acyclovir	B	III		
Patients with MPN, treated with ruxolitinib	To reduce HSV disease	Acyclovir	C	IIu		[85–89]
	To prevent herpes zoster^b^	Acyclovir	B	IIru		[86–88]

In patients with normal renal function, acyclovir is recommended with 400 mg orally BID and valacyclovir is recommended with 500 mg orally BID (for more details refer to section “Pharmacological Prophylaxis).

*AML* acute myeloid leukaemia, *ALL* acute lymphoblastic leukaemia, *APL* acute promyelocytic leukaemia, *HSV* herpes simplex virus, *MDS* myelodysplastic syndrome, *MPN* myeloproliferative neoplasm, *QoE* quality of evidence, *SoR* strength of recommendation, *VZV* varicella zoster virus.

^a^Valacyclovir may be used as well, although trials are limited compared to acyclovir (see text).

^b^Data are mainly available for herpes zoster; evidence for other clinical reactivations of VZV is unclear.

Data on VZV reactivation rates are sparse, reported only in few trials [33, 80–82], but they seem low during the time of intensive chemotherapy. However, a very recent retrospective trial pointed at the significant risk of VZV reactivation in patients with acute promyelocytic leukaemia treated with arsenic trioxide: In 112 patients, disease by reactivation of VZV occurred in 17.5% of patients (including one patient with VZV encephalitis) without versus in 4.1% of patients with prophylaxis with acyclovir or valacyclovir (RR 0.24) [83]. Therefore, pharmacological prophylaxis for this patient group during the time of treatment till 6 months thereafter is recommended to reduce VZV disease (BIIr) (Table 6).

Only two older trials [78, 81] within the meta-analysis by Yahav et al. [33] reported about patients with acute lymphoblastic leukaemia. As these patients are exposed to even more intensive and prolonged chemotherapy protocols, including corticosteroids and sometimes anti-CD20 monoclonal antibodies (see below “Patients with lymphoma”) [84], antiviral prophylaxis to reduce reactivation of HSV and VZV is recommended for patients with acute lymphoblastic leukaemia while on treatment (BI) (Table 6).

### Patients with myeloproliferative neoplasm

Infectious complications are main causes of morbidity and mortality in patients with myeloproliferative neoplasms, particularly in patients with myelofibrosis in advanced stage. Moreover, ruxolitinib, an inhibitor auf Janus kinases, modulates dendritic cell function resulting in impaired CD4 and CD8 T cell priming [85]. A systematic meta-analysis including five phase III trials on ruxolitinib revealed higher rates of herpes zoster among patients treated with ruxolitinib compared to control patients (OR 5.20) [86]. A retrospective study published as abstract showed similar results with an OR for VZV/HSV reactivation of 7.57 [87]. Results of registry data confirm the clinical relevance of clinical herpesvirus reactivations, mainly as herpes zoster [88, 89], but the benefit of antiviral prophylaxis has not been prospectively or retrospectively validated [89]. Recommendations and QoE are depicted in Table 6. While some authors [85, 89] favour the implementation of antiviral prophylaxis, high awareness by patient and doctor, thorough clinical examination and immediate treatment of reactivations, is generally warranted.

### Patients with lymphoma

There are few data systematically analysing the risk of HSV and VZV reactivations in lymphoma patients. Moreover, to the best of our knowledge, results from recent randomized controlled trials on antiviral pharmacological prophylaxis have not been published. Two retrospective analyses from Korea evaluated the incidence of disease by herpesvirus reactivations in patients with lymphoma not receiving antiviral prophylaxis: Park et al. [90] found an incidence of herpesvirus disease of 10.7% in 270 patients with diffuse large B cell lymphoma (DLBCL) within 30 months of receiving immuno-chemotherapy with R-CHOP, of which 75.9% were caused by VZV, 20.7% by HSV, and 3.4% by CMV. Lee et al. [4] described the cumulative incidence of herpesvirus disease in 266 patients with non-Hodgkin lymphoma and Hodgkin’s lymphoma at 5 years being 20.16%. Again, reactivations of VZV were dominating with 93% [4]. Both trials [4, 90] identified a high cumulative dose of corticosteroids (cumulative PEQ dose ≥ 2500 mg/m^2^ or 3000 mg/m^2^ body surface area (BSA)) and a history of neutropenic fever as independent risk factors on multivariate analysis. Whereas these retrospective data only describe incidences of clinical reactivations of *herpesviridae*, introducing oral acyclovir (400 mg QID given from the start of therapy until four weeks after the last therapy cycle) together with cotrimoxazol (2 double strength doses twice a week) in addition to oral ciprofloxacin (500 mg BID) as anti-infective prophylaxis in the OPTIMAL > 60-trial significantly reduced grade 3/4 infections of all kind (from 28 to 18% per patient, p = 0.004) and treatment-related mortality (from 7 to 2%, p = 0.003) in elderly patients with DLBCL compared to a historical control [91]. Antiviral prophylaxis was also shown to reduce the risk of clinical reactivations of VZV and HSV in patients with indolent lymphomas treated with bendamustine ± anti-CD20 monoclonal antibody [92]. This evidence supports the use of pharmacological prophylaxis to reduce particularly VZV disease in non-Hodgkin lymphoma patients treated with (immuno-)chemotherapy (BIIu) (Table 7). Patients planned to receive > 2500 mg/m^2^ BSA PEQ dose may especially benefit because risk of clinical reactivation of herpesvirus is high [4, 90], furthermore age > 60 years, advanced line of therapy, treatment with bendamustine, history of febrile neutropenia and history of HSV/VZV reactivation have been identified as risk factors.

**Table 7:** Patients with lymphoma, chronic lymphocytic leukaemia and multiple myeloma

Clinical situation	Intention	Intervention	SoR	QoE	Comments	Reference
Patients with non-Hodgkin lymphoma, treated with immuno-chemotherapy^a^	To reduce HSV/VZV disease	Acyclovir (valacyclovir)^b^	B	IIu	Persisting risk for several months after therapy (see text)	[4, 90, 92, 96, 130, 131]
	To reduce mortality	Acyclovir	C	IIah	Together with cotrimoxazol, in patients aged > 60 years	[91]
Patients with Hodgkin’s disease^a^	To prevent herpes zoster	Acyclovir (valacyclovir) ^b)^	C	III		[89]
Patients with CLL receiving immuno-chemotherapy^a^	To reduce HSV/VZV disease	Acyclovir, (valacyclovir) ^b)^	B	IIuh	Persisting risk for several months after therapy (see text)	[96–99]
Patients with CLL (and other Non- Hodgkin lymphoma) receiving BTK or BCL2 inhibitors^a^	To prevent herpes zoster (to reduce VZV/HSV disease)	Acyclovir, (valacyclovir)^b^	C	IIu	Of benefit particularly in advanced lines of therapy	[103–107, 109, 110, 132]
Patients with CLL (and other Non-Hodgkin lymphoma) receiving idelalisib	to reduce HSV/VZV disease	acyclovir	B	III	High general risk of opportunistic infections, persisting for several months after therapy	[111, 113, 133]
Patients with MM, receiving bortezomib	To reduce VZV disease^c^	Acyclovir, valacyclovir	A B	IIu IIu	^d^	[62, 64, 118–120, 125, 134–137]
Patient with MM receiving carfilzomib	To reduce VZV disease^c^	e.g., acyclovir	A	IIu	^d^	[117, 119, 122, 127]
Patients with MM receiving ixazomib	To reduce VZV disease^c^	e.g., acyclovir	A	IIh	^d^	[121, 123]
Patients with MM receiving lenalidomid	To reduce VZV disease^c^	e.g. acyclovir	C	IIh	^d^	[121, 124]
Patients with MM receiving daratumumab	To reduce VZV disease^c^	e.g. acyclovir	C	IIt	^d^	[116, 117, 128]
Patients with MM receiving elotuzumab	To reduce VZV disease^c^	e.g. acyclovir	C	IIt	^d^	[117, 138]
Patients with MM receiving conventional-dose chemotherapy^e^ or other targeted agents^a^	To reduce VZV disease^c^	e.g. acyclovir	C	IIt	^d^	[117, 126] (see also above)

In patients with normal renal function, acyclovir is recommended with 400 mg orally BID or QID and valacyclovir is recommended with 500 mg orally BID (details are given in the section “Pharmacological Prophylaxis”).

*CLL* chronic lymphocytic leukaemia, *MM* multiple myeloma, *BTK* Bruton’s tyrosine kinase, *BCL2* B-cell-lymphoma kinase 2, *HSCT* haematopoietic stem cell transplantation, *HSV* herpes simplex virus, *QoE* quality of evidence, *SoR* strength of recommendation, *VZV* varicella zoster virus.

^a^Individual risk assessment is recommended: The following risk factors have been described in patients with non-Hodgkin lymphoma or CLL: age > 60 years, concomitant treatment with high doses of corticosteroids (cumulative PEQ dose > 2500 mg/m^2^ BSA), advanced line of therapy, type of therapy (bendamustine, maintenance by anti-CD20 monoclonal antibodies), history of febrile neutropenia, and history of HSV/VZV reactivation. The risk factors may also help in decision making for antiviral prophylaxis in patients with multiple myeloma.

^b^Valacyclovir has been used as well, but evidence is less clear.

^c^VZV disease: data mainly refer to herpes zoster. Data on HSV disease are rarely reported (see text).

^d^Reactivation risk by a single agent is difficult to determine, because combinations were mainly used; prophylaxis in trials was frequently open (“might be considered or recommended”), and duration of prophylaxis has not been determined.

^e^Excluding patients with multiple myeloma treated with high-dose chemotherapy and autologous HSCT. For those antiviral pharmacological prophylaxis is highly recommended: A IIt (to prevent HSV reactivation) and A IIu (to prevent VZV reactivation), details in [8]

There is no general recommendation for antiviral prophylaxis in patients with first line therapy of Hodgkin’s lymphoma (treated with ABVD or BEACOPPesc) according to study protocols [93, 94]. Still the risk of VZV reactivation may be elevated, partly due to corticosteroid exposure (Table 5). Decision about antiviral prophylaxis has to be made on individual case basis, referring to treatment intensity and duration (CIII) (Table 7). Advanced lines of therapy may increase the risk of clinical HSV/VZV reactivation [89].

Antiviral prophylaxis is most effective during the first year after starting treatment for lymphoma. But reactivations occurred up to 51.3 months from initial immuno-chemotherapy [4], particularly in patients treated with rituximab plus bendamustine and in patients with rituximab or obinutuzumab maintenance [92]. Therefore the duration of antiviral prophylaxis may be extended according to individual risk assessment. SoR and QoE for patients with non-Hodgkin lymphoma and Hodgkin’s disease are summarized in Table 7.

### Patients with chronic lymphocytic leukaemia

Patients with chronic lymphocytic leukaemia (CLL) are at increased risk for infection because of compromised immune function, which might be related to the disease itself and/or to the therapy [29, 95]. In a recent retrospective analysis, published as abstract [96], the risk for herpes zoster was comparable in 1210 multiple myeloma patients and 2520 CLL patients (10.5% and 12.9% respectively). In a Swedish nationwide analysis from 1994 to 2013 of inpatient opportunistic infectious complications in CLL patients, herpes zoster was second most common after pneumocystis jirovecii pneumonia. Incidence rates per 1000 patient-years were 2.94 for herpes zoster and 0.8 for HSV reactivations, compared to 0.26 and 0.04 respectively in the age-/sex-/residence-matched control group [97]. Only inpatients were included, thus patients with severe disease, which was also reflected by an early mortality rate of 15% (for herpes zoster) and 13% (for HSV reactivation) [97]. A decreasing rate of herpes zoster cases during time course was possibly linked to increasing use of antiviral pharmacological prophylaxis. However, there are no general recommendations for antiviral pharmacological prophylaxis in patients with CLL [95], probably because the patient population as well as treatment modalities are heterogeneous and the efficacy of antiviral pharmacological prophylaxis has not been evaluated in randomized controlled trials. Thus, for patients with CLL treated with immuno-chemotherapy, our recommendations do not differ from the recommendations for lymphoma patients (BIIuh) (Table 7). Special consideration has to be taken to patients treated with rituximab plus bendamustine or rituximab in combination with fludarabine and cyclophosphamide, as herpesvirus reactivation has been reported frequently and up to 3 years after treatment [98, 99]. Additionally elderly patients, patients with advanced lines of therapy, and patients with a history of VZV or HSV reactivation [100] have been described to be at higher risk and may benefit particularly of pharmacological prophylaxis (Table 7).

Ibrutinib binds covalently Bruton’s tyrosine kinase (BTK) and inhibits its enzymatic activity on development and activity of B-lymphocytes and leads to depression of serum gamma globulins [29, 101]. Concerning herpesvirus reactivations, case reports have demonstrated the potential of fatal disseminated varicella zoster with BTK-inhibition [31, 32, 102]. In contrast, increased opportunistic infections in the ibrutinib-treated cohorts in four phase III trials [103–106] were not seen, while only one trial required antiviral prophylaxis in both arms [103]. Coutre et al. [107] described herpes zoster and oral herpes disease in 5% and 4% respectively during long-term follow-up of 330 patients of the RESONATE and RESONATE-2 trial [105, 108]. We could therefore not find evidence supporting a general recommendation for antiviral prophylaxis in patients treated with BTK inhibitors. Clinicians should be aware that patients may develop atypical infectious complications, including severe reactivations of VZV and HSV. Antiviral prophylaxis might be implemented on the patient’s individual risk, e.g. in case of high age or advanced line of therapy (CIIu) (Table 7).

Intracellular signalling of B-Cell-Lymphoma kinase 2 (BCL2) promotes cellular survival and is highly overexpressed in CLL and other malignancies. Venetoclax is a small molecule binding BCL2 specifically, leading to rapid and deep remissions in CLL patients. Importantly, first line treatment with venetoclax in combination with obinutuzumab lead to grade 3/4 neutropenia in 52.8% and grade 3/4 infections in 17.5% of patients in the pioneering CLL-14 trial [109]. These adverse events did not differ significantly from the control group treated with obinutuzumab plus chlorambucil [109]. Seymour et al. [110] observed comparable adverse events when treating patients with relapsed or refractory CLL with rituximab together with venetoclax. Of note, rates of febrile neutropenia and severe infectious complications were lower in the rituximab plus venetoclax group than in the control group treated with rituximab plus bendamustine [110]. Viral infectious complications have not been reported separately. There was no mandatory antimicrobial prophylaxis in neither of the studies. The existing data about venetoclax are not sufficient to consider a specific risk for VZV or HSV reactivations. In summary, antiviral pharmacological prophylaxis should be administered on the basis of the patient´s individual risk as described above (CIIu) (Table 7).

The use of idelalisib, a phosphatidylinositol-3-kinase-pathway (PI3Kδ)-inhibitor, has been associated with fatal opportunistic infections [111] and relatively frequent reactivations of CMV [112]. For this reason, antiviral pharmacological prophylaxis has been used widely [113] — limiting data on reactivation rates of HSV and VZV [114] — and is recommended (BIII) (Table 7). Moreover, close monitoring for infectious complications is indicated.

Hypogammaglobulinemia is a well-recognized complication associated with CLL. Regarding CLL patients with hypogammaglobulinemia (IgG below 4 g/L) and history of infections, different randomized studies have shown that the prophylactic use of intravenous immunoglobulins decreases the rate of bacterial infections, but not of non-bacterial infections [115]. There is no evidence for a beneficial effect specific on herpesvirus reactivations by polyclonal immunoglobulin substitution.

### Patients with multiple myeloma

Reactivation by VZV has been described as frequent infectious complication in patients with multiple myeloma [116, 117] and mainly presents as herpes zoster. It is an important clinical issue in patients with multiple myeloma in all phases of active disease. The proteasome inhibitor bortezomib, in combination with either corticosteroids or other drugs such as melphalan or daratumumab, substantially increases the risk for herpes zoster [62, 116, 117]. Several, mainly retrospective reports indicate that acyclovir prophylaxis significantly reduces the risk of herpes zoster in these patients [118, 119]. The risk of herpes zoster was 13% in the cohort receiving bortezomib and 4% in the control arm in the VISTA trial analysing melphalan-prednisolone with or without bortezomib [62]. The incidence of herpes zoster was reduced to 3% in patients getting bortezomib together with antiviral prophylaxis. Therefore, we strongly recommend acyclovir prophylaxis in patients receiving bortezomib-based treatment regimens (AII) (Table 7). Alternatively valacyclovir might be used [63, 64, 119, 120] (BIIu) (Table 7). More limited data are available on herpes zoster risk and prophylactic strategies in patients receiving other proteasome inhibitors such as carfilzomib or ixazomib [121–123]. Patients who started VZV prophylaxis at study entry had a significantly lower herpes zoster incidence (≤ 1%) in a randomized controlled trial comparing lenalidomide-dexamethasone with ixazomib (versus placebo) in comparison to those not starting VZV prophylaxis (8% and 3% in the ixazomib versus placebo arm, respectively) [121, 123]. Therefore, the increased VZV reactivation risk after proteasome inhibitor-based regimens is rather class-related than associated with selected agents. As a consequence, we also recommend the use of antiviral prophylaxis, mainly with acyclovir, in patients receiving proteasome inhibitors other than bortezomib (AIIu; AIIh) (Table 7). The VZV reactivation risk seems to be rather low (< 5% in the majority of controlled trials) in patients with multiple myeloma receiving other targeted agents (e.g. antibodies, immunomodulatory drugs (IMiDs)), and there are limited data on the use of antiviral prophylaxis in these patients [116, 124, 125]. In summary, we recommend their use only in selected cases, taking in account the patient’s individual VZV disease risk [124] (CIIh; CIIt) (Table 7).

HSV reactivation as stomatitis, if seen, predominantly affects patients treated with autologous HSCT. Recommendations on the management of infections after autologous HSCT — including prophylaxis in multiple myeloma patients — have been published by the AGIHO recently [8]. Limited data exist on the HSV reactivation risk and antiviral prophylaxis in the current era of multiple myeloma management outside the HSCT setting [20, 124, 126–128]. HSV reactivation was reported in 2.7% of patients in an integrated safety profile analysis of single-agent carfilzomib in 526 patients included in four phase II trials [127]. Hereby, antiviral medication was administered concomitantly to 63% of patients while being on study. Taken together, antiviral prophylaxis might be used with the aim to reduce the risk of HSV disease patients with multiple myeloma on an individual case basis (Table 7).

## Treatment

Decision for treatment is made after considering the diagnosis on clinical grounds, before (and sometimes without) it is confirmed by virus detection (see Diagnostics). Treatment modalities depend on (a) type and severity of HSV or VZV reactivation, (b) the clinical condition of the patient, and (c) severity of immunosuppression. Oral administration can be used if disease is localized, symptoms are minor, and the immunosuppression can be considered as mild. In these cases, valacyclovir (1000 mg TID) and famciclovir (500 mg BID or TID respectively for herpes genitalis or herpes zoster) may be alternatives to oral acyclovir (400 mg five times per day for localised HSV disease or 800 mg five times per day for herpes zoster), with the limitation that data are deduced from the immunocompromised cohort of patients with human immunodeficiency virus (HIV). Therapy lasts 7 to 10 days in most cases. While on therapy, close clinical monitoring is essential to switch early to intravenous acyclovir when signs of cutaneous dissemination, involvement of the central nervous system, or other organs occur [139]. In patients with severe immunosuppression and thus a high risk for complications, upfront intravenous therapy should be considered [11, 52]. Careful clinical decision making is necessary as early effective therapy is important to reduce complication rate. For intravenous therapy, acyclovir is used with 5 mg/kg body weight TID for HSV reactivation by localized disease [38] and 10 mg/kg body weight TID for disseminated, cerebral or visceral disease of HSV and VZV and for herpes zoster. Disseminated disease as well as cerebral and visceral disease requires treatment for at least 14 days.

At large, treatment recommendations for patients with malignancies are consistent with recommendations and guidelines for the general population [19, 24, 52, 140–143] as trials specific for patients with malignancies have not been performed. It has to be noticed, though, that brivudin is not approved in the immunocompromised patients and most importantly contraindicated in patients treated with 5-fluoruracil or its prodrugs (like capecitabine, tegafur) due to potential lethal hematologic toxicity [25, 38, 144]. Further information about treatment in the immunocompromised patient can also be derived from the AGIHO guideline on infections of the central nervous system in patients with hematological disorders [49] and from the S2-consensus-based guideline, referring to patients after solid organ transplantation or allogeneic HSCT [38].

## Conclusion

This guideline updates our recommendations on antiviral prophylaxis in patients with solid tumours and hematological malignancies of 2015 [9]. As it is relevant for some strategies and an often raised question in clinical practice, we included recommendations on diagnostics.

HSV stomatitis (in patients with leukaemia treated with intensive chemotherapy) and herpes zoster (in patients with lymphoma and multiple myeloma) are the most frequent clinical herpesvirus reactivations and affect a considerable number of patients. Prophylaxis with acyclovir (or valacyclovir) has been successfully implemented to reduce these reactivations in different situations, while insufficient data still exist about the effect of antiviral prophylaxis on severe HSV or VZV disease and mortality. Since the previous publication of our guideline, only few prospective randomized studies have been published on prophylaxis of HSV und VZV. But evidence originating from retrospective trials and registries has increased substantially. Recent developments in herpes zoster vaccination strategies have led attention on VZV reactivation rates and clinical sequelae of herpes zoster. Incidence rates from large population groups are now available to better assess relative risk in different groups of patient with malignancies. We therefore update our recommendations from 2015 with regard to different patient groups. Because increasing data show the considerable risk of disease by reactivations of HSV or VZV in specific patient populations, the recommendation was often upgraded. Vaccination strategies will most likely influence the risk of VZV reactivations in the future. Up to now, some data exist on clinical outcomes, mainly in patients with hematologic malignancies, and immunological efficacy (with variable results). Further research is necessary on vaccination efficacy (short- and long-term) in different anticancer treatments.

## Data Availability

Not applicable.

## Abbreviations

AGIHO: Infectious Diseases Working Party
ASCO: American Society of Oncology
ASH: American Society of Hematology
BAL: Bronchoalveolar lavage
BCL2: B-cell-lymphoma kinase 2
BID: Twice daily
BSA: Body surface area
BTK: Bruton’s tyrosine kinase
BW: Bronchial wash
CI: Confidence interval
CMV: Cytomegalovirus
CSF: Cerebrospinal fluid
CT: Computed tomography
DGHO: German Society for Hematology and Medical Oncology
DLBCL: Diffuse large B-cell lymphoma
DNA: Desoxyribonucleic acid
EHA: European Hematology Association (EHA)
EMA: European Medicines Agency
ESCMID: European Society for Clinical Microbiology and Infectious Diseases
ESMO: European Society of Medical Oncology
EBV: Epstein-Barr virus
HHV-6: Human herpesvirus type 6
HIV: Human immunodeficiency virus
HSCT: Hematopoietic stem cell transplantation
HSV-1: Herpes simplex virus type 1
HSV-2: Herpes simplex virus type 2
ISOO: International Society of Oral Oncology
MASCC: Multinational Association of Supportive Care in Cancer
MRI: Magnetic resonance imaging
qPCR: Quantitative polymerase chain reaction
QID: Four times daily
QoE: Quality of evidence
RCT: Randomized controlled trial
RR: Relative risk
SoR: Strength of recommendation
TID: Three times daily
VZV: Varicella zoster virus

## References

[1] Chen S-Y, Suaya JA, Li Q, Galindo CM, Misurski D, Burstin S (2014). Incidence of herpes zoster in patients with altered immune function. Infection.

[2] Ruiz-Camps I, Aguilar-Company J (2021). Risk of infection associated with targeted therapies for solid organ and hematological malignancies. Ther Adv Infect Dis.

[3] Teh BW, Harrison SJ, Worth LJ, Thursky KA, Slavin MA (2016). Infection risk with immunomodulatory and proteasome inhibitor-based therapies across treatment phases for multiple myeloma: a systematic review and meta-analysis. Eur J Cancer.

[4] Lee HS, Park JY, Shin SH, Kim SB, Lee JS, Lee A (2012). Herpesviridae viral infections after chemotherapy without antiviral prophylaxis in patients with malignant lymphoma: incidence and risk factors. Am J Clin Oncol.

[5] Tan IL, McArthur JC, Venkatesan A, Nath A (2012). Atypical manifestations and poor outcome of herpes simplex encephalitis in the immunocompromised. Neurology.

[6] Styczynski J (2021). Management of herpesvirus infections in hematopoietic cell transplant recipients. Transplantology.

[7] Beyar-Katz O, Bitterman R, Zuckerman T, Ofran Y, Yahav D, Paul M (2020). Anti-herpesvirus prophylaxis, pre-emptive treatment or no treatment in adults undergoing allogeneic transplant for haematological disease: systematic review and meta-analysis. Clin Microbiol Infect.

[8] Christopeit M, Schmidt-Hieber M, Sprute R, Buchheidt D, Hentrich M, Karthaus M (2020). Prophylaxis, diagnosis and therapy of infections in patients undergoing high-dose chemotherapy and autologous haematopoietic stem cell transplantation. 2020 update of the recommendations of the Infectious Diseases Working Party (AGIHO) of the German Society of Hematology and Medical Oncology (DGHO). Ann Hematol.

[9] Sandherr M, Hentrich M, von Lilienfeld-Toal M, Massenkeil G, Neumann S, Penack O (2015). Antiviral prophylaxis in patients with solid tumours and haematological malignancies–update of the Guidelines of the Infectious Diseases Working Party (AGIHO) of the German Society for Hematology and Medical Oncology (DGHO). Ann Hematol.

[10] Ullmann AJ, Akova M, Herbrecht R, Viscoli C, Arendrup MC, Arikan-Akdagli S (2012). ESCMID* guideline for the diagnosis and management of Candida diseases 2012: adults with haematological malignancies and after haematopoietic stem cell transplantation (HCT). Clin Microbiol Infect.

[11] Levin MJ, Weinberg A, Schmid DS (2016). Herpes simplex virus and varicella-zoster virus. Microbiol Spectrum.

[12] Glenny A-M, Fernandez Mauleffinch LM, Pavitt S, Walsh T (2009). Interventions for the prevention and treatment of herpes simplex virus in patients being treated for cancer. Cochrane Database Syst Rev.

[13] Herget GW, Riede UN, Schmitt-Gräff A, Lübbert M, Neumann-Haefelin D, Köhler G (2005). Generalized herpes simplex virus infection in an immunocompromised patient–report of a case and review of the literature. Pathol Res Pract.

[14] Djuric M, Jankovic L, Jovanovic T, Pavlica D, Brkic S, Knezevic A (2009). Prevalence of oral herpes simplex virus reactivation in cancer patients: a comparison of different techniques of viral detection. J Oral Pathol Med.

[15] Spruance SL, Bodsworth N, Resnick H, Conant M, Oeuvray C, Gao J (2006). Single-dose, patient-initiated famciclovir: a randomized, double-blind, placebo-controlled trial for episodic treatment of herpes labialis. J Am Acad Dermatol.

[16] Christman MP, Turbett SE, Sengupta S, Bakhadirov KU, Williamson CA, Nayak L (2014). Recurrence of herpes simplex encephalitis associated with temozolomide chemoradiation for malignant glioma: a case report and review of the literature. Oxf Med Case Reports.

[17] Graber JJ, Rosenblum MK, DeAngelis LM (2011). Herpes simplex encephalitis in patients with cancer. J Neurooncol.

[18] Toomey DP, Dhadda AS, Sanni LA, Cooke JP, Hartley JE (2014). Fatal herpes simplex virus hepatitis following neoadjuvant chemoradiotherapy and anterior resection for rectal cancer. Ann R Coll Surg Engl.

[19] Patel R, Kennedy OJ, Clarke E, Geretti A, Nilsen A, Lautenschlager S (2017). 2017 European guidelines for the management of genital herpes. Int J STD AIDS.

[20] Hong J, Park H-K, Park S, Lee A, Lee Y-H, Shin D-Y (2020). Strong association between herpes simplex virus-1 and chemotherapy-induced oral mucositis in patients with hematologic malignancies. Korean J Intern Med.

[21] Whitley RJ, Kimberlin DW, Roizman B (1998). Herpes simplex viruses. Clin Infect Diseases.

[22] Le Cleach L, Trinquart L, Do G, Maruani A, Lebrun-Vignes B, Ravaud P (2014). Oral antiviral therapy for prevention of genital herpes outbreaks in immunocompetent and nonpregnant patients. Cochrane Database Syst Rev.

[23] Gupta R, Wald A, Krantz E, Selke S, Warren T, Vargas-Cortes M (2004). Valacyclovir and Acyclovir for suppression of shedding of herpes simplex virus in the genital tract. J Infect Dis.

[24] Gershon AA, Breuer J, Cohen JI, Cohrs RJ, Gershon MD, Gilden D (2015). Varicella zoster virus infection. Nat Rev Dis Primers.

[25] Andrei G, Snoeck R (2021). Advances and perspectives in the management of varicella-zoster virus infections. Molecules.

[26] Dagnew AF, Ilhan O, Lee W-S, Woszczyk D, Kwak J-Y, Bowcock S (2019). Immunogenicity and safety of the adjuvanted recombinant zoster vaccine in adults with haematological malignancies: a phase 3, randomised, clinical trial and post-hoc efficacy analysis. Lancet Infect Dis.

[27] Lal H, Cunningham AL, Godeaux O, Chlibek R, Diez-Domingo J, Hwang S-J (2015). Efficacy of an adjuvanted herpes zoster subunit vaccine in older adults. N Engl J Med.

[28] Habel LA, Ray GT, Silverberg MJ, Horberg MA, Yawn BP, Castillo AL (2013). The epidemiology of herpes zoster in patients with newly diagnosed cancer. Cancer Epidemiol Biomarkers Prev.

[29] Williams AM, Baran AM, Meacham PJ, Feldman MM, Valencia HE, Newsom-Stewart C (2018). Analysis of the risk of infection in patients with chronic lymphocytic leukemia in the era of novel therapies. Leuk Lymphoma.

[30] van der Beek MT, Vermont CL, Bredius RGM, Marijt EWA, van der Blij-de Brouwer CS, Kroes ACM (2013). Persistence and antiviral resistance of varicella zoster virus in hematological patients. Clin Infect Diseases.

[31] Giridhar KV, Shanafelt T, Tosh PK, Parikh SA, Call TG (2017). Disseminated herpes zoster in chronic lymphocytic leukemia (CLL) patients treated with B-cell receptor pathway inhibitors. Leuk Lymphoma.

[32] Sutton E, Lopez JJ, Dao LN, Wetter DA (2016). Disseminated herpes zoster in chronic lymphocytic leukemia. J Emerg Med.

[33] Yahav D, Gafter-Gvili A, Muchtar E, Skalsky K, Kariv G, Yeshurun M (2009). Antiviral prophylaxis in haematological patients: systematic review and meta-analysis. Euro J Cancer.

[34] Elad S, Ranna V, Ariyawardana A, Correa MEP, Tilly V, Nair RG (2017). A systematic review of oral herpetic viral infections in cancer patients: commonly used outcome measures and interventions. Support Care Cancer.

[35] Angarone M (2011). Epidemiology and prevention of viral infections in patients with hematologic malignancies. Infect Disord Drug Targets.

[36] Aribi Al-Zoobaee FW, Yee SL, Veettil SK, Gopinath D, Maharajan MK, Menon RK (2020). Antiviral agents for the prevention and treatment of herpes simplex virus type-1 infection in clinical oncology: a network meta-analysis. Int J Environ Res Public Health.

[37] Taplitz RA, Kennedy Erin B, Bow Eric J, Crews J, Gleason C, Hawley DK (2018). Antimicrobial prophylaxis for adult patients with cancer-related immunosuppression: ASCO and IDSA Clinical Practice Guideline Update. J Clin Oncol.

[38] Koordinatorin: Schmidt B. Virusinfektionen bei Organ- und allogen Stammzell-Transplantierten: Diagnostik, Prävention und Therapie: S2k-Leitlinie. AWMF Registernummer 093–002. [November 30, 2020]; Available from: https://www.awmf.org/leitlinien/detail/ll/093-002.html.

[39] Wald A, Huang ML, Carrell D, Selke S, Corey L (2003). Polymerase chain reaction for detection of herpes simplex virus (HSV) DNA on mucosal surfaces: comparison with HSV isolation in cell culture. J Infect Dis.

[40] Levin MJ (2014). Varicella-zoster virus and virus DNA in the blood and oropharynx of people with latent or active varicella-zoster virus infections. J Clin Virol.

[41] Wilson DA, Yen-Lieberman B, Schindler S, Asamoto K, Schold JD, Procop GW (2012). Should varicella-zoster virus culture be eliminated? A comparison of direct immunofluorescence antigen detection, culture, and PCR, with a historical review. J Clin Microbiol.

[42] Bhullar SS, Chandak NH, Purohit HJ, Taori GM, Daginawala HF, Kashyap RS (2014). Determination of viral load by quantitative real-time PCR in herpes simplex encephalitis patients. Intervirology.

[43] van der Beek MT, Laheij AMGA, Raber-Durlacher JE, von dem Borne PA, Wolterbeek R, van der Blij-de Brouwer CS (2012). Viral loads and antiviral resistance of herpesviruses and oral ulcerations in hematopoietic stem cell transplant recipients. Bone Marrow Transplant.

[44] Sauerbrei A (2016). Herpes genitalis: diagnosis, treatment and prevention. Geburtshilfe Frauenheilkd.

[45] Park SY, Kim JY, Kim J-A, Kwon J-S, Kim S-M, Jeon NY (2017). Diagnostic usefulness of varicella-zoster virus real-time polymerase chain reaction analysis of DNA in saliva and plasma specimens from patients with herpes zoster. J INFECT DIS.

[46] Gershon AA (2011). The history and mystery of VZV in saliva. J INFECT DIS.

[47] Ramchandani M, Kong M, Tronstein E, Selke S, Mikhaylova A, Magaret A (2016). Herpes simplex virus type 1 shedding in tears and nasal and oral mucosa of healthy adults. Sex Transm Dis.

[48] Stram MN, Suciu CN, Seheult JN, McCullough MA, Kader M, Wells A (2018). Herpes simplex virus-1 qPCR in the diagnosis of lower respiratory tract infections in organ transplant recipients and critically ill patients. Am J Clin Pathol.

[49] Schmidt-Hieber M, Silling G, Schalk E, Heinz W, Panse J, Penack O (2016). CNS infections in patients with hematological disorders (including allogeneic stem-cell transplantation)-Guidelines of the Infectious Diseases Working Party (AGIHO) of the German Society of Hematology and Medical Oncology (DGHO). Ann Oncol.

[50] Schuierer L, Gebhard M, Ruf H-G, Jaschinski U, Berghaus TM, Wittmann M (2020). Impact of acyclovir use on survival of patients with ventilator-associated pneumonia and high load herpes simplex virus replication. Crit Care.

[51] Luyt C-E, Forel J-M, Hajage D, Jaber S, Cayot-Constantin S, Rimmelé T (2020). Acyclovir for mechanically ventilated patients with herpes simplex virus oropharyngeal reactivation: a randomized clinical trial. JAMA Intern Med.

[52] Gross GE, Eisert L, Doerr HW, Fickenscher H, Knuf M, Maier P (2020). S2k-Leitlinie zur Diagnostik und Therapie des Zoster und der Postzosterneuralgie. GMS Infect Dis.

[53] Grahn A, Bergström T, Runesson J, Studahl M (2016). Varicella-zoster virus (VZV) DNA in serum of patients with VZV central nervous system infections. J Infect.

[54] Mirouse A, Vignon P, Piron P, Robert R, Papazian L, Géri G (2017). Severe varicella-zoster virus pneumonia: a multicenter cohort study. Crit Care.

[55] Ishizaki Y, Tezuka J, Ohga S, Nomura A, Suga N, Kuromaru R (2003). Quantification of circulating varicella zoster virus-DNA for the early diagnosis of visceral varicella. J Infect.

[56] Sahoo F, Hill JA, Xie H, Leisenring W, Yi J, Goyal S (2017). Herpes zoster in autologous hematopoietic cell transplant recipients in the era of acyclovir or valacyclovir prophylaxis and novel treatment and maintenance therapies. Biol Blood Marrow Transplant.

[57] Baumrin E, Cheng MP, Kanjilal S, Ho VT, Issa NC, Baden LR (2019). Severe herpes zoster requiring intravenous antiviral treatment in allogeneic hematopoietic cell transplantation recipients on standard acyclovir prophylaxis. Biol Blood Marrow Transplant.

[58] van der Beek MT, Claas ECJ, van der Blij-de Brouwer CS, Morfin F, Rusman LG, Kroes ACM (2013). Rapid susceptibility testing for herpes simplex virus type 1 using real-time PCR. J Clin Virol.

[59] Steingrimsdottir H, Gruber A, Kalin M, Eksborg S (2000). Bioavailability of aciclovir after oral administration of aciclovir and its prodrug valaciclovir to patients with leukopenia after chemotherapy. Antimicrob Agents Chemother.

[60] Warkentin DI, Epstein JB, Campbell LM, Yip JG, Cox VC, Ransier A (2002). Valacyclovir versus acyclovir for HSV prophylaxis in neutropenic patients. Ann Pharmacother.

[61] Lee CJ, Savani BN, Ljungman P (2018). Varicella zoster virus reactivation in adult survivors of hematopoietic cell transplantation: how do we best protect our patients?. Biol Blood Marrow Transplant.

[62] San Miguel JF, Schlag R, Khuageva NK, Dimopoulos MA, Shpilberg O, Kropff M (2008). Bortezomib plus melphalan and prednisone for initial treatment of multiple myeloma. N Engl J Med.

[63] Fukushima T, Sato T, Nakamura T, Iwao H, Nakajima A, Miki M (2012). Daily 500 mg valacyclovir is effective for prevention of Varicella zoster virus reactivation in patients with multiple myeloma treated with bortezomib. Anticancer Res.

[64] Usami E, Kimura M, Iwai M, Teramachi H, Yoshimura T (2016). Prophylactic efficacy against herpes zoster and costs difference between acyclovir and valaciclovir in hematological patients. In Vivo.

[65] Bastidas A, de La Serna J, El Idrissi M, Oostvogels L, Quittet P, López-Jiménez J (2019). Effect of recombinant zoster vaccine on incidence of herpes zoster after autologous stem cell transplantation: a randomized clinical trial. JAMA.

[66] Vink P, Delgado Mingorance I, Maximiano Alonso C, Rubio-Viqueira B, Jung KH, Rodriguez Moreno JF (2019). Immunogenicity and safety of the adjuvanted recombinant zoster vaccine in patients with solid tumors, vaccinated before or during chemotherapy: a randomized trial. Cancer.

[67] Zent CS, Brady MT, Delage C, Strawderman M, Laniewski N, Contant PN (2020). Short term results of vaccination with adjuvanted recombinant varicella zoster glycoprotein E during initial BTK inhibitor therapy for CLL or lymphoplasmacytic lymphoma. Leukemia.

[68] Pleyer C, Ali MA, Cohen JI, Tian X, Soto S, Ahn IE (2021). Effect of Bruton tyrosine kinase inhibitor on efficacy of adjuvanted recombinant hepatitis B and zoster vaccines. Blood.

[69] Ljungman P (2019). Varicella zoster virus vaccine in patients with haematological malignancies. Lancet Infect Dis.

[70] Ilyas S, Chandrasekar PH (2020). Preventing varicella-zoster: advances with the recombinant zoster vaccine. Open Forum Infect Dis.

[71] Sakoh T, Kanzaki M, Miyamoto A, Mochizuki S, Kakumoto T, Sato K (2019). Ramsay-Hunt syndrome and subsequent sensory neuropathy as potential immune-related adverse events of nivolumab: a case report. BMC Cancer.

[72] Watanabe Y, Kikuchi R, Iwai Y, Ito M, Tsukamoto H, Yamazaki K (2019). Varicella zoster virus encephalitis mimicking nivolumab-induced autoimmune neuropathy in a patient with lung cancer. J Thorac Oncol.

[73] Sumer J, Waldeck F, Fischer N, Appenzeller C, Koster M, Früh M (2021). HSV-pneumonitis in a patient with lung cancer receiving check point inhibitors — a case report. Pneumonia (Nathan).

[74] Del Castillo M, Romero FA, Argüello E, Kyi C, Postow MA, Redelman-Sidi G (2016). The spectrum of serious infections among patients receiving immune checkpoint blockade for the treatment of melanoma. Clin Infect Dis.

[75] Ramirez-Fort MK, Zeng J, Feily A, Ramirez-Pacheco LA, Jenrette JM, Mayhew DL (2018). Radiotherapy-induced reactivation of neurotrophic human herpes viruses: overview and management. J Clin Virol.

[76] Malpica L, Moll S (2020). Practical approach to monitoring and prevention of infectious complications associated with systemic corticosteroids, antimetabolites, cyclosporine, and cyclophosphamide in nonmalignant hematologic diseases. Hematol Am Soc Hematol Educ Program.

[77] Fardet L, Petersen I, Nazareth I (2016). Common infections in patients prescribed systemic glucocorticoids in primary care: a population-based cohort study. PLoS Med.

[78] Anderson H, Scarffe JH, Sutton RN, Hickmott E, Brigden D, Burke C (1984). Oral acyclovir prophylaxis against herpes simplex virus in non-Hodgkin lymphoma and acute lymphoblastic leukaemia patients receiving remission induction chemotherapy. A randomised double blind, placebo controlled trial. Brit J Cancer.

[79] Bergmann OJ, Ellermann-Eriksen S, Mogensesn SC, Ellegaard J (1995). Acyclovir given as prophylaxis agienst oral ulcers in acute myeloid leukaemia: randomised, double blind, placebo controlled trial. BMJ.

[80] Hann IM, Prentice HG, Blacklock HA, Ross MG, Brigden D, Rosling AE (1983). Acyclovir prophylaxis against herpes virus infections in severly immunocompromised patients: randomised double blind trial. Br Med J (Clin REs Ed).

[81] Lönnqvist B, Palmblad J, Ljungman P, Grimfors G, Järnmark M, Lerner R (1993). Oral acyclovir as prophylaxis for bacterial infections during induction therapy for acute leukaemia in adults. The Leukemia Group of Middle Sweden. Support Care Cancer.

[82] Saral R, Ambinder RF, Burns WH, Angelopulos CM, Griffin DE, Burke PJ (1983). Acyclovir prophylaxis against herpes simplex virus infection in patients with leukemia. A randomized, double-blind, placebo-controlled study. Ann Intern Med.

[83] Freyer CW, Peterson CE, Man Y, Przespolewski A, Baron J, Luger SM (2021). Herpes zoster during arsenic trioxide therapy for acute promyelocytic leukemia. Leuk Lymphoma.

[84] Logan C, Koura D, Taplitz R (2020). Updates in infection risk and management in acute leukemia. Hematol Am Soc Hematol Educ Program.

[85] Heine A-C, Brossart P, Wolf D (2013). Ruxolitinib is a potent immunosuppressive compound: is it time for anti-infective prophylaxis?. Blood.

[86] Lussana F, Cattaneo M, Rambaldi A, Squizzato A (2018). Ruxolitinib-associated infections: a systematic review and meta-analysis. Am J Hematol.

[87] Gauthier N, Soret-Dulphy J, Zhao L-P, De Daltro OR, Marcault C, Parquet N (2020). Ruxolitinib treatment is associated with increased incidence of infections and higher risk of HSV/VZV recurrence in patients with myeloproliferative neoplasm (MPN) related myelofibrosis (MF). Blood.

[88] Polverelli N, Palumbo GA, Binotto G, Abruzzese E, Benevolo G, Bergamaschi M (2018). Epidemiology, outcome, and risk factors for infectious complications in myelofibrosis patients receiving ruxolitinib: a multicenter study on 446 patients. Hematol Oncol.

[89] Maschmeyer G, de Greef J, Mellinghoff SC, Nosari A, Thiebaut-Bertrand A, Bergeron A, et al. (2019) Infections associated with immunotherapeutic and molecular targeted agents in hematology and oncology. A position paper by the European Conference on Infections in Leukemia (ECIL). Leukemia 33(4):844–862. 10.1038/s41375-019-0388-x. 10.1038/s41375-019-0388-x PMC6484704 30700842

[90] Park LC, Lee HS, Shin SH, Im H, Ye BJ, Song MK (2011). Herpesviridae viral infections following rituximab combined chemotherapy in patients with diffuse large B-cell lymphoma. Acta Haematol.

[91] Murawski N, Amam J, Altmann B, Ziepert M, Haenel M, Viardot A (2017). Anti-infective prophylaxis with aciclovir and cotrimoxazole significantly reduces the rate of infections and therapyassociated deaths in elderly patients with DLBCL undergoing R-CHOP immunochemotherapy. Oncol Res Treatment.

[92] Manos K, Lasica M, Grigg A, Di Ciaccio PR, Wong J, Chandra Sekaran U (2020). Prolonged lymphopenia and infection risk is mitigated by antimicrobial prophylaxis in patients with indolent non-hodgkin lymphoma (iNHL) treated with bendamustine +/- anti-CD20 antibody: the Australasian lymphoma alliance experience. Blood.

[93] Gillessen S, Plütschow A, Fuchs M, Markova J, Greil R, Topp MS (2021). Intensified treatment of patients with early stage, unfavourable Hodgkin lymphoma: long-term follow-up of a randomised, international phase 3 trial of the German Hodgkin Study Group (GHSG HD14). Lancet Haematol.

[94] Borchmann P, Plütschow A, Kobe C, Greil R, Meissner J, Topp MS (2021). PET-guided omission of radiotherapy in early-stage unfavourable Hodgkin lymphoma (GHSG HD17): a multicentre, open-label, randomised, phase 3 trial. Lancet Oncol.

[95] Hallek M, Cheson BD, Catovsky D, Caligaris-Cappio F, Dighiero G, Döhner H (2018). iwCLL guidelines for diagnosis, indications for treatment, response assessment, and supportive management of CLL. Blood.

[96] Hussaini SB, Chu J, Williams N, Sharma N, Benson Don M, Rosko AE (2017). B-cell lymphoma patients have a 10% risk of shingles, 7% in cutaneous T-cell lymphoma. Blood.

[97] Steingrímsson V, Gíslason GK, Þorsteinsdóttir S, Rögnvaldsson S, Gottfreðsson M, Aspelund T (2021). A nationwide study on inpatient opportunistic infections in patients with chronic lymphocytic leukemia in the pre-ibrutinib era. Eur J Haematol.

[98] Allen JM, Rybicki LA, Caimi PF, Jagadeesh D, Dean RM, Pohlman B (2015). Standard duration of varicella zoster virus prophylaxis may be inadequate to prevent viral reactivation in patients with chronic lymphocytic leukemia treated with front-line bendamustine plus rituximab. Blood.

[99] Srivastava R, Griswold D, Jamil MO (2018). Chronic lymphocytic leukaemia with necrotic herpetic adenitis: an elusive clinical condition. BMJ Case Rep.

[100] Oscier D, Dearden C, Eren E, Fegan C, Follows G, Hillmen P (2012). Guidelines on the diagnosis, investigation and management of chronic lymphocytic leukaemia. Br J Haematol.

[101] Tillman BF, Pauff JM, Satyanarayana G, Talbott M, Warner JL (2018). Systematic review of infectious events with the Bruton tyrosine kinase inhibitor ibrutinib in the treatment of hematologic malignancies. Eur J Haematol.

[102] Chen I, Fohtung RB, Oughli HA, Bauer R, Mattar C, Powderly WG (2016). Concurrent Ramsay Hunt syndrome and disseminated herpes zoster in a patient with relapsed chronic lymphocytic leukemia. IDCases.

[103] Shanafelt TD, Wang XV, Kay NE, Hanson CA, O'Brien S, Barrientos J (2019). Ibrutinib-rituximab or chemoimmunotherapy for chronic lymphocytic leukemia. N Engl J Med.

[104] Moreno C, Greil R, Demirkan F, Tedeschi A, Anz B, Larratt L (2019). Ibrutinib plus obinutuzumab versus chlorambucil plus obinutuzumab in first-line treatment of chronic lymphocytic leukaemia (iLLUMINATE): a multicentre, randomised, open-label, phase 3 trial. Lancet Oncol.

[105] Burger JA, Tedeschi A, Barr PM, Robak T, Owen C, Ghia P (2015). Ibrutinib as initial therapy for patients with chronic lymphocytic leukemia. N Engl J Med.

[106] Woyach JA, Ruppert AS, Heerema NA, Zhao W, Booth AM, Ding W (2018). Ibrutinib regimens versus chemoimmunotherapy in older patients with untreated CLL. N Engl J Med.

[107] Coutre SE, Byrd JC, Hillmen P, Barrientos JC, Barr PM, Devereux S (2019). Long-term safety of single-agent ibrutinib in patients with chronic lymphocytic leukemia in 3 pivotal studies. Blood Adv.

[108] Byrd JC, Brown JR, O'Brien S, Barrientos JC, Kay NE, Reddy NM (2014). Ibrutinib versus ofatumumab in previously treated chronic lymphoid leukemia. N Engl J Med.

[109] Fischer K, Al-Sawaf O, Bahlo J, Fink A-M, Tandon M, Dixon M (2019). Venetoclax and obinutuzumab in patients with CLL and coexisting conditions. N Engl J Med.

[110] Seymour JF, Kipps TJ, Eichhorst B, Hillmen P, D'Rozario J, Assouline S (2018). Venetoclax- rituximab in relapsed or refractory chronic lymphocytic leukemia. N Engl J Med.

[111] Zelenetz AD, Barrientos JC, Brown JR, Coiffier B, Delgado J, Egyed M (2017). Idelalisib or placebo in combination with bendamustine and rituximab in patients with relapsed or refractory chronic lymphocytic leukaemia: interim results from a phase 3, randomised, double-blind, placebo-controlled trial. Lancet Oncol.

[112] Cheah CY, Fowler NH (2016). Idelalisib in the management of lymphoma. Blood.

[113] Hanlon A, Brander DM (2020). Managing toxicities of phosphatidylinositol-3-kinase (PI3K) inhibitors. Hematol Am Soc Hematol Educ Program.

[114] Bird ST, Tian F, Flowers N, Przepiorka D, Wang R, Jung T-H (2020). Idelalisib for treatment of relapsed follicular lymphoma and chronic lymphocytic leukemia: a comparison of treatment outcomes in clinical trial participants vs medicare beneficiaries. JAMA Oncol.

[115] Sánchez-Ramón S, Dhalla F, Chapel H (2016). Challenges in the role of gammaglobulin replacement therapy and vaccination strategies for hematological malignancy. Front Immunol.

[116] Drgona L, Gudiol C, Lanini S, Salzberger B, Ippolito G, Mikulska M (2018). ESCMID Study Group for Infections in Compromised Hosts (ESGICH) Consensus Document on the safety of targeted and biological therapies: an infectious diseases perspective (Agents targeting lymphoid or myeloid cells surface antigens II: CD22, CD30, CD33, CD38, CD40, SLAMF-7 and CCR4). Clin Microbiol Infect.

[117] Ludwig H, Delforge M, Facon T, Einsele H, Gay F, Moreau P (2018). Prevention and management of adverse events of novel agents in multiple myeloma: a consensus of the European Myeloma Network. Leukemia.

[118] Minarik J, Pika T, Bacovsky J, Langova K, Scudla V (2012). Low-dose acyclovir prophylaxis for bortezomib-induced herpes zoster in multiple myeloma patients. Br J Haematol.

[119] Leng S, Lentzsch S, Shen Y, Tsai W-Y, Wright JD, Hershman DL (2018). Use and impact of herpes zoster prophylaxis in myeloma patients treated with proteasome inhibitors. Leuk Lymphoma.

[120] Vickrey E, Allen S, Mehta J, Singhal S (2009). Acyclovir to prevent reactivation of varicella zoster virus (herpes zoster) in multiple myeloma patients receiving bortezomib therapy. Cancer.

[121] Moreau P, Masszi T, Grzasko N, Bahlis NJ, Hansson M, Pour L (2016). Oral ixazomib, lenalidomide, and dexamethasone for multiple myeloma. N Engl J Med.

[122] Muchtar E, Gertz MA, Magen H (2016). A practical review on carfilzomib in multiple myeloma. Eur J Haematol.

[123] Kumar S, Moreau P, Hari P, Mateos M-V, Ludwig H, Shustik C (2017). Management of adverse events associated with ixazomib plus lenalidomide/dexamethasone in relapsed/refractory multiple myeloma. Br J Haematol.

[124] König C, Kleber M, Reinhardt H, Knop S, Wäsch R, Engelhardt M (2014). Incidence, risk factors, and implemented prophylaxis of varicella zoster virus infection, including complicated varicella zoster virus and herpes simplex virus infections, in lenalidomide-treated multiple myeloma patients. Ann Hematol.

[125] Teh BW, Harrison SJ, Worth LJ, Slavin MA (2016). Antiviral prophylaxis for varicella zoster virus infections in patients with myeloma in the era of novel therapies. Leuk Lymphoma.

[126] Nucci M, Anaissie E (2009). Infections in patients with multiple myeloma in the era of high-dose therapy and novel agents. Clin Infect Diseases.

[127] Siegel D, Martin T, Nooka A, Harvey RD, Vij R, Niesvizky R (2013). Integrated safety profile of single-agent carfilzomib: experience from 526 patients enrolled in 4 phase II clinical studies. Haematologica.

[128] Nahi H, Chrobok M, Gran C, Lund J, Gruber A, Gahrton G (2019). Infectious complications and NK cell depletion following daratumumab treatment of Multiple Myeloma. PLoS ONE.

[129] Bergmann OJ, Mogensen SC, Ellermann-Eriksen S, Ellegaard J (1997). Acyclovir prophylaxis and fever during remission-induction therapy of patients with acute myeloid leukemia: a randomized, double-blind, placebo-controlled trial. J Clin Oncol.

[130] Dang T, Ni A, Gerecitano JF, Hamlin PA, Hohl TM, Kumar A (2016). Incidence of infectious complications associated with bendamustine and anti-CD20 monoclonal antibody combination at Memorial Sloan Kettering Cancer Center (MSKCC). Blood.

[131] Vazquez GY, Rivera Concepcion J, Cabanillas F (2018). Incidence and risk factors for developing herpes zoster among a cohort of patients diagnosed with lymphoma at a community cancer center. Blood.

[132] Stephens DM, Byrd JC (2019). How I manage ibrutinib intolerance and complications in patients with chronic lymphocytic leukemia. Blood.

[133] Attallah J, Khan U, Sharma M, Kafri Z (2019). Disseminated herpes simplex virus type 2 after treatment with idelalisib. BJSTR.

[134] Chanan-Khan A, Sonneveld P, Schuster MW, Stadtmauer EA, Facon T, Harousseau J-L (2008). Analysis of herpes zoster events among bortezomib-treated patients in the phase III APEX study. J Clin Oncol.

[135] Pour L, Adam Z, Buresova L, Krejci M, Krivanova A, Sandecka V (2009). Varicella-zoster virus prophylaxis with low-dose acyclovir in patients with multiple myeloma treated with bortezomib. Clin Lymphoma Myeloma.

[136] Kim SJ, Kim K, Do YR, Bae SH, Yang D-H, Lee J-J (2011). Low-dose acyclovir is effective for prevention of herpes zoster in myeloma patients treated with bortezomib: a report from the Korean Multiple Myeloma Working Party (KMMWP) Retrospective Study. Jpn J Clin Oncol.

[137] Swaika A, Paulus A, Miller KC, Sher T, Almyroudis NG, Ball D (2012). Acyclovir prophylaxis against varicella zoster virus reactivation in multiple myeloma patients treated with bortezomib-based therapies: a retrospective analysis of 100 patients. J Support Oncol.

[138] Dimopoulos MA, Dytfeld D, Grosicki S, Moreau P, Takezako N, Hori M (2018). Elotuzumab plus pomalidomide and dexamethasone for multiple myeloma. N Engl J Med.

[139] Nikkels AF, Piérard GE (2002). Oral antivirals revisited in the treatment of herpes zoster: what do they accomplish?. Am J Clin Dermatol.

[140] Werner RN, Nikkels AF, Marinović B, Schäfer M, Czarnecka-Operacz M, Agius AM (2017). European consensus-based (S2k) guideline on the management of herpes zoster — guided by the European Dermatology Forum (EDF) in cooperation with the European Academy of Dermatology and Venereology (EADV), Part 2: Treatment. J Eur Acad Dermatol Venereol.

[141] Bradshaw MJ, Venkatesan A (2016). Herpes simplex virus-1 encephalitis in adults: pathophysiology, diagnosis, and management. Neurotherapeutics.

[142] Kalezic T, Mazen M, Kuklinski E, Asbell P (2018). Herpetic eye disease study: lessons learned. Curr Opin Ophthalmol.

[143] Li JY (2018). Herpes zoster ophthalmicus: acute keratitis. Curr Opin Ophthalmol.

[144] Rätz Bravo AE, Hofer S, Krähenbühl S, Ludwig C (2009). Fatal drug-drug interaction of brivudine and capecitabine. Acta Oncol.

